# Hidradenitis Suppurativa: Molecular Etiology, Pathophysiology, and Management—A Systematic Review

**DOI:** 10.3390/cimb45050280

**Published:** 2023-05-17

**Authors:** Michael Joseph Diaz, Shaliz Aflatooni, Parsa Abdi, Rina Li, Michelle Robert Anthony, Sphurti Neelam, Chris Farkouh, Jasmine Thuy Tran, Steven Svoboda, Mahtab Forouzandeh, Rodrigo H. Valdes Rodriguez

**Affiliations:** 1 College of Medicine, University of Florida, Gainesville, FL 32610, USA; michaeldiaz@ufl.edu (M.J.D.);; 2 Morsani College of Medicine, University of South Florida, Tampa, FL 33602, USA; 3 Faculty of Medicine, Memorial University, St. Johns, NL A1B 3V6, Canada; 4 Department of Sociology, Amherst College, Amherst, MA 01002, USA; 5 College of Medicine, The University of Arizona, Tucson, AZ 85724, USA; 6 Rush Medical College, Rush University, Chicago, IL 60612, USA; 7 School of Medicine, University of Indiana, Indianapolis, IN 46202, USA; 8 Department of Dermatology, University of Florida College of Medicine, Gainesville, FL 32606, USA

**Keywords:** hidradenitis suppurativa, acne inversa, etiology, pathophysiology, treatment, systematic review

## Abstract

Hidradenitis suppurativa is a chronic inflammatory skin condition that affects the hair follicles in areas of the body with apocrine glands. The condition is characterized by recurrent, painful nodules, abscesses, and draining sinuses that can lead to scarring and disfigurement. In this present study, we provide a focused evaluation of recent developments in hidradenitis suppurativa research, including novel therapeutics and promising biomarkers that may facilitate clinical diagnosis and treatment. We conducted a systematic review of controlled trials, randomized controlled trials, meta-analyses, case reports, and Cochrane Review articles in accordance with the PRISMA guidelines. The Cochrane Library, PubMed, EMBASE, and Epistemonikos databases were queried via Title/Abstract screen. Eligibility criteria included the following: (1) has a primary focus on hidradenitis suppurativa, (2) includes measurable outcomes data with robust comparators, (3) details the sample population, (4) English language, and (5) archived as full-text journal articles. A total of 42 eligible articles were selected for review. Qualitative evaluation identified numerous developments in our understanding of the disease’s multiple potential etiologies, pathophysiology, and treatment options. It is important for individuals with hidradenitis suppurativa to work closely with a healthcare provider to develop a comprehensive treatment plan that addresses their individual needs and goals. To meet this objective, providers must keep current with developments in the genetic, immunological, microbiological, and environmental factors contributing to the disease’s development and progression.

## 1. Introduction

Hidradenitis suppurativa (HS), also known as acne inversa, is a chronic inflammatory skin disorder characterized by the development of persistent or recurrent double-headed comedones, painful, firm papules and nodules, draining sinuses linking inflammatory lesions, and residual hypertrophic and atrophic scarring. HS lesions most commonly manifest in the intertriginous and apocrine gland-rich regions, namely the axillary, groin, perianal, perineal, and inframammary regions. However, HS can occur in any skin region containing a follicular portion of folliculopilosebaceous units [1]. Cysts and abscesses are a possible result of follicular occlusion, which occurs when hair follicle apertures narrow [2]. This early pathogenic event causes follicular rupture and subsequent inflammation. Numerous factors, such as excessive oil production and bacterial infections of the scalp, may lead to the formation of HS. A link between HS and three diseases that display this histological principle (pilonidal sinus, dissecting cellulitis of the scalp, and acne conglobata) supports this possible disease mechanism [2]. Similarly, in some circumstances, friction from tight clothing or shaving can cause hair follicles to swell or become irritated. A blocked hair follicle can result in the buildup of fluid and germs, which can cause the follicle to rupture and spread the infection to the tissues surrounding it. These symptoms, including inflammation, malodor, drainage, and local deformity, significantly impact the quality of life for many patients, with HS being linked to an increased risk for cardiovascular events, completed suicides, and all-cause mortality [3,4]. HS typically presents during puberty or early adulthood affecting an estimated 1–4% of the general population, with a female predominance [5].

Although there is currently no cure for HS, treatment options are available depending on the severity of the disease [6]. The current treatment modalities include topical and systemic antibiotics, anti-inflammatory agents, biologics, hormonal therapy, and surgical interventions, such as marsupialization and excision of the affected skin. However, not every patient is a candidate for surgical interventions, which have been shown to significantly enhance quality of life, and the efficacy of systemic therapies is limited in a significant population of patients, leading to a need for a better understanding of the underlying pathophysiology of the disease [7,8,9].

The exact cause of HS is unknown. However, it is thought to be a multifaceted disease caused by genetic, environmental, and immune system components [1]. HS may be linked to abnormal immune activation that results in persistent inflammation and skin barrier malfunction, with obesity, smoking, and hormone abnormalities being significant risk factors [10]. Despite the expanding knowledge of HS pathophysiology, there is a substantial gap in the literature covering recent developments in HS research, emphasizing the necessity for carefully assessing the existing literature. This systematic review aims to evaluate and appraise the current literature on the pathophysiology, etiology, and treatment of HS, specifically focusing on recent developments in HS research, including potential novel therapeutic options and promising biomarkers that may facilitate disease diagnosis and monitoring.

## 2. Methods

### 2.1. Study Design

A systematic review of controlled trials, randomized controlled trials (RCTs), meta-analyses, case reports, and Cochrane Review articles was conducted in accordance with the latest Preferred Reporting Items for Systematic Reviews and Meta-Analyses (PRIMSA) guidelines [11]. This review was registered in the international prospective register of systematic review (PROSPERO) (CRD42023410111).

### 2.2. Search Strategy

The Cochrane Library, PubMed, EMBASE, and Epistemonikos databases were broadly queried on 10 March 2023 to retrieve all relevant articles since the database’s inception. The main keyword search terms were (“hidradenitis suppurativa” OR “acne inversa”) AND (“etiology” OR “pathophysiology” OR “treatment”). Queries were restricted to the Title and Abstract fields (“[tiab]”). Search records were maintained with Covidence, a web-based collaborative platform for conducting systematic reviews [12].

### 2.3. Eligibility Criteria

All initial search results were subjected to the following inclusion criteria: (1) has a primary focus on hidradenitis suppurativa; (2) includes measurable outcomes data, with robust comparators (i.e., changes compared to baseline or comparison to age-matched, healthy human patients or non-exposed controls); (3) includes a number of human subjects or cell line type, where applicable. Criteria for exclusion were (1) non-English articles, (2) abstract-only text, and (3) not yet-published clinical trials.

### 2.4. Data Extraction

**Figure 1:**
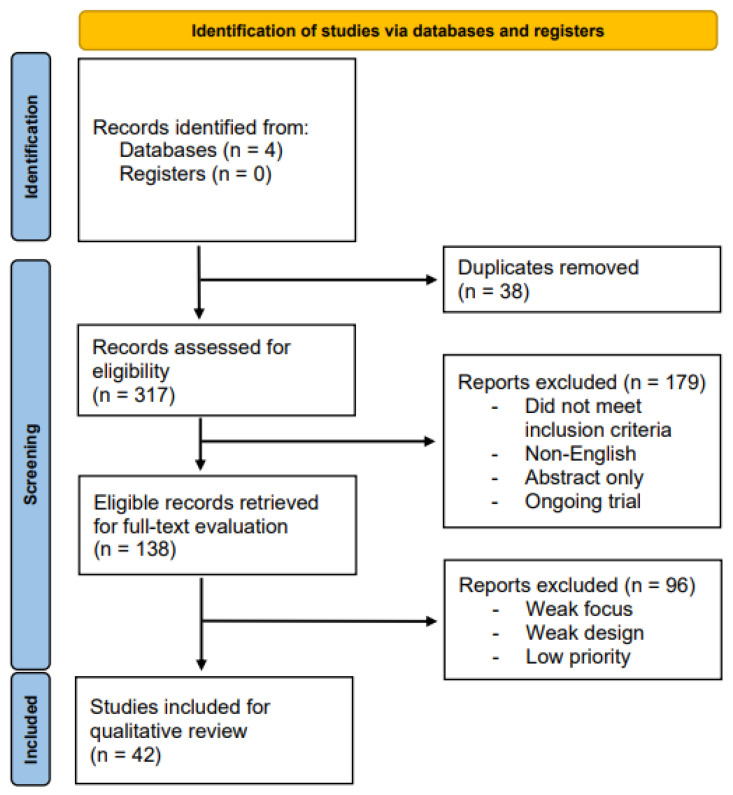
PRISMA flow diagram.

The initial sensitivity search returned 317 total records. Following the elimination of duplicates, 279 identified articles underwent independent abstract and title screening for eligibility assessment by two of the authors (JTT, PA) using the Covidence systematic review software. From these records, 138 articles were deemed eligible for full-text review. After a thorough examination of the relevant studies, 39 studies were deemed suitable for qualitative synthesis. Any discrepancies in the selection process were resolved through discussion or by seeking the input of an impartial third party (MJD). Full-text consultation of the eligible article set for topic relevance, results novelty, and methodology robustness narrowed the final selection to 36 articles. Figure 1 depicts the PRISMA selection process flowchart used for this systematic review.

## 3. Results

### 3.1. Etiology

HS is a chronic inflammatory disorder that affects apocrine gland-bearing skin in the axillae, groin, and under the breasts [13]. Severe pain, inflammatory secretion, immobility, and systemic participation brought on by HS all contribute to a reduced quality of life. Etiologies of HS can be broken down into environmental, genetic, and hormonal factors. Table 1 summarizes nine articles that have been selectively chosen for their relevance in elucidating the etiology of HS.

#### 3.1.1. Environmental Correlation

Smoking and obesity are lifestyle choices that add to the development of HS. These variables cause plugging and follicle rupture by altering the microbiome, causing subclinical inflammation around the terminal hair follicles and inducing infundibular hyperkeratosis [14]. The ratio of sex-specific smoking rates in North America, Europe, and Asia is strongly correlated with the sex ratio in individuals with hidradenitis suppurativa [15]. It has also been proposed that psychological tension contributes to the emergence and progression of HS. Stress can alter immune functions, resulting in disease onset or exacerbation [16]. Additionally, stress and anxiety can lead to behaviors such as smoking or binge eating that exacerbate the symptoms of HS.

#### 3.1.2. Genetic Correlation

A hereditary component with an autosomal dominant transmission pattern is suggested by the fact that 33 to 40 percent of people with hidradenitis suppurativa describe a first-degree relative with the condition [17]. In a small population of individuals affected with HS, an altered microbiome may cause an overabundance of anaerobic opportunistic bacteria and biofilm development with an abnormal immune response. Examples of genetic factors that have been associated with HS include alterations in the genes for interleukin (IL)-1, IL-12, and IL-23, as well as mutations in the gamma-secretase genes [17]. The latter are involved in the Notch signaling pathway, which plays a crucial role in skin development. Similar to this, HS has been linked to variations in the genes that encode IL-12 and IL-23, two cytokines implicated in the adaptive immune response [18]. These cytokines have been found to be increased in HS lesions and are involved in the differentiation and activation of T cells.

#### 3.1.3. Hormonal Correlation

The development of HS is more common in women and may be influenced by endogenous hormone secretion. Androgens, especially testosterone, have been linked to HS because they promote apocrine gland growth and secretion [19]. Choi et al. revealed that low serum zinc and vitamin D levels are associated with increased lesion count in HS, suggesting that vitamins may play a role in the pathogenesis of the disease [19]. Molinelli and colleagues reported that supplementation with 90 mg of zinc gluconate corresponded with a reduced number and duration of acute flareups [20]. Finally, results from six case-control studies, based on unadjusted analysis, indicated a significant association between adult cases of HS and metabolic syndrome, furthering the support of hormones’ role in HS [21].

### 3.2. Pathophysiology

The pathophysiology of HS involves a complicated interplay of various processes, including follicular hyperkeratosis, sweat gland dysfunction, immune dysregulation, bacterial infection, and chronic inflammation. Together, these factors contribute to inflammatory and tissue damage characteristics of the disease. Table 2 summarizes the thirteen selected articles reporting on the pathophysiology of hidradenitis suppurativa.

As one of the “follicular occlusion tetrad”, HS is thought to have pathophysiology initiated by the blockage of the follicular portion of folliculopilosebaceous units (FPSU) [19]. Follicular hyperkeratosis occurs when a hair follicle opening in the gland-bearing areas of the body becomes thickened and blocked, which then progresses to follicle rupture [22]. Studies have proven that the inflammation of HS was connected to abnormalities in skin regulation pathways, namely extracellular matrix (ECM) organization, keratinization, cornified envelope formation, epidermal cell differentiation, and collagen formation and degradation [22]. Elafin (PI3), an elastase-specific inhibitor expressed by epithelial and some immune cells to act as an antimicrobial peptide, was reported to have eight times higher expression. SERPINs, a broad family of serine protease inhibitors, were also overexpressed in HS patients’ hair follicles [22]. Their upregulation and overexpression suggest the connection between HS and keratinocyte hyperproliferation. The keratinization and formation of the cornified envelope may be attributed to the dysregulation of certain cytokeratin, including KRT6-KRT16 and SPRRs proteins. Separately, the upregulation of 12 types of matrix metalloproteinases (MMPs) possibly creates a long-term inflammatory response in HS-damaged skin, as this extracellular protease continues to break down and remodel the ECM [22]. Lastly, several genes, namely *WIF1*, *AQP5*, *FOXA1*, and *DCD*, responsible for regulating sweat glands’ proper functioning were found to be significantly less expressed in HS skin compared to healthy skin, compromising the skin’s ability to repair and re-epithelialize [22]. Another study also characterizes HS with the dysregulation of the γ-secretase/Notch pathway [17]. The decline in the Notch signaling pathways causes hair follicles’ transformation into keratin-enriched epidermal cysts, undermining apocrine gland homeostasis. A lack of Notch signaling also leads to the stimulation of toll-like receptor-mediated innate immunity, sustaining chronic inflammation. The immune system responds by increasing the secretion of TNF-α and IL-1β, which activates dendritic cells. Dendritic cells in turn release IL-23, leading to Th17 cell polarization and increasing IL-17 production [17]. Other studies also supported that HS was partially mediated by the overexpression of TNF-α [22,23,24]. Prior review deemed TNF-α as the major cytokine in the pathophysiology of HS, as its overexpression in both lesional and perilesional skin of HS had a positive correlation with disease severity [17]. The upregulation of proinflammatory cytokines such as IL-17, IL-23, IL-1β, and TNF-α activated by the proliferation of macrophages further perpetuates skin inflammation [22,25].

Despite the precise underlying mechanisms remaining unknown, previous studies posit that factors such as genetic susceptibility, immune dysfunction, and cytokine dysregulation may have contributed to the comorbidities between HS and inflammatory arthritis, bowel diseases, allergic diseases, stroke, thyroid disease, and renal diseases [18,26,27,28,29]. Almuhanna et al. [26] reported that compared to 385,599 controls, the 200,361 HS patients had a significantly higher risk of inflammatory arthritis (OR 3.44; 95% CI 1.92–6.17), the association between HS and spondyloarthritis (OR 2.10; 95% CI 1.40–3.15), and the association between HS and rheumatoid arthritis (OR 1.89; 95% CI 1.14–3.12). A review by Gau et al. [27] found that based on 12 studies with 196,757 participants, HS patients have a 1.50-fold greater prevalence of asthma (95% CI 1.24–1.81) and a higher risk of developing atopic dermatitis (OR 4.10; 95% CI 2.06–8.18). In another review, Gau [28] found that in five observational studies involving 402,021 participants, HS patients had a 2.63-fold increased risk of renal diseases (95% CI 2.03–3.42). Phan et al. [29] reported in their review of six case-control studies that HS patients reported a significantly greater proportion of strokes than the control ((OR 1.74; 95% CI 1.45–2.09; *p* < 0.00001). Lastly, another review of five case-control studies found that 36,103 HS cases had a significant association with thyroid disease (OR 1.36; 95% CI 1.13–1.64, *p* = 0.001), compared to the 170,517 control cases. Furthermore, the impact of HS is not only physical but also psychological. The high disease burden of HS also increases the odds of depression and other psychiatric symptoms, such as lower self-esteem and greater levels of anger and emotional fragility [16,30].

### 3.3. Current Treatment Opportunities

HS is a complex disease that can present a significant challenge for patients and clinicians to find the optimal and effective treatment, leading to increased morbidity and suffering. Over 50 treatments have been attempted, including topical, systemic, and surgical interventions [31]. Clinicians ultimately assess HS severity through Hurley staging to determine the best treatment options for patients. See Table 3 for a summary of 10 selected articles reporting on current treatment options for hidradenitis suppurativa.

#### 3.3.1. Topical Regimens

For mild HS, topical treatments are frequently employed, with systematic therapies and surgical approaches subsequently added with increased extension and severity of HS lesions [14]. Common topical antibiotics used for HS include clindamycin, erythromycin, and gentamicin [32]. Topical antibiotics such as clindamycin have been shown to decrease HS lesions through a 3-month randomized controlled trial but were limited by the exclusion of HS staging [13]. Other topical treatments used in this setting include benzoyl peroxide wash, retinoids, and resorcinol [32].

#### 3.3.2. Oral Antibiotics

For widespread or refractory HS, oral antibiotics are often considered. Common treatment regimens include tetracyclines, clindamycin monotherapy, clindamycin + rifampicin, clindamycin + ofloxacin, trimethoprim/sulfamethoxazole, dapsone, and ertapenem [32]. A frequent oral regime used includes 500 mg of tetracycline used twice a day for a period of between 12 and 16 weeks [32]. A randomized control trial demonstrated no significant change in HS lesions with oral tetracycline compared to topical clindamycin [13]. Additionally, 300 mg of clindamycin with or without 300 mg of rifampicin was the second preferred regime with tetracycline failure [32]. A disadvantage of using the rifampicin and clindamycin combination is that rifampicin is a CYP-450 3A4 inducer, which can significantly decrease clindamycin levels. Furthermore, rifampicin increases the risk of bacterial resistance, signifying the importance of alternative therapies such as biologicals in the treatment of HS. The combination of bacterial resistance to rifampicin and decreased clindamycin levels increases the risk of treatment failure with the above combination. Delaunay et al. performed a retrospective study involving a combination of ofloxacin and clindamycin compared to rifampicin–clindamycin for the treatment of HS [33]. They found that combining ofloxacin and clindamycin improved disease activity in 58.4% of individuals. This data suggests that ofloxacin and clindamycin may be potential alternative treatments for HS. Still, it is essential to recognize the need for further prospective studies. Their analysis was limited by severity being assessed according to the number of flares pre-and post-treatment and the small sample size [33]. Oral retinoids have also been used to treat HS, including acitretin and isotretinoin [32]. Common antiandrogens used include metformin, finasteride, and ethinylestradiol [32].

#### 3.3.3. Biologic Agents and Immunomodulatory Drugs

Various biologics and immunomodulators are used and researched for HS. TNF inhibitors have been increasingly employed in the treatment of HS. An FDA-approved TNF inhibitor for moderate-to-severe HS includes adalimumab. Unlike oral antibiotics, these biologics are used for the long term to provide maintenance control [32]. PIONEER I and PIONEER II were clinical trials that demonstrated Adalimumab’s effectiveness in treating HS [32]. Marasca et al. performed a single-center retrospective study that compared rifampicin and clindamycin antibiotic combination to adalimumab for HS presenting with moderate to severe manifestations. The study found that rifampicin, the clindamycin antibiotic combination, and adalimumab decreased the mean modified Sartorius Score, but the reduction was more significantly decreased with adalimumab [34]. A chimeric mouse/human anti-TNF monoclonal antibody used as a second-line biological for HS includes infliximab. It is often used when Hurley stage III HS resists adalimumab therapy [32]. Shih et al. conducted a systematic review and meta-analysis involving the treatment of moderate to severe HS with infliximab. They found an 83% pooled response rate of HS to infliximab, highlighting infliximab’s effectiveness [35]. 

Moreover, the IL-12/23 pathway has been implicated in HS pathogenesis. A human monoclonal antibody for IL-12 and IL-23, ustekinumab, has been used off-label in patients with severe HS who failed treatment with adalimumab and infliximab [32]. Romaní et al. performed a multicentric retrospective study on ustekinumab to treat HS. They showed moderate efficacy for treating HS, as shown by the Dermatology Life Quality Index (DLQI) and visual analog scale (VAS) of pain improvement in 71.42% of ustekinumab-treated patients. However, the study was limited by a small sample size and a shorter period to follow up [36]. Blok and colleagues performed an open-label study involving 17 patients treated with ustekinumab. They found that ustekinumab improved HS symptomatology in most individuals, as shown by 82% of patients having a moderate-to-marked improvement of the modified Sartorius score by 40 weeks of treatment [37]. IL-17A pathway has also been implicated in HS pathogenesis, and secukinumab monoclonal antibody against IL-17A has shown to be effective in patients with severe HS [32]. Thorlacius et al. presents a case report involving a 47-year-old male diagnosed with Hurley stage III HS with diffuse involvement who had failed multiple therapeutic approaches [38]. He was treated with secukinumab for 12 weeks and found to have a significant decrease in the number of HS lesions, pain visual analog scale (VAS) score, and pain/utility/handicap VAS score [38]. Two prominent phase 3 trials, SUNSHINE and SUNRISE, reported that secukinumab given every two weeks resulted in rapid improvement of HS symptoms and 52 weeks of sustained response to secukinumab [39].

Other immune-modulating agents, including apremilast, etanercept, rituximab, anakinra, canakinumab, and certolizumab pegol, have been utilized for HS with mixed results. Apremilast is a phosphodiesterase four inhibitor given in an oral form that was studied for treating mild to moderate HS in an open-label, phase 2 clinical trial. It was found that Hidradenitis Suppurativa Clinical Response 30 (HiSCR30) was successfully accomplished in 65% of patients, and the most common symptoms, diarrhea, nausea, and depression, were found in 20%, 15%, and 10%, respectively [40]. Overall, the study showed that apremilast is both effective and safe for HS with mild to moderate severity and may be a potential option for HS patients with a less severe presentation [40]. New biological drugs being investigated for HS include bermekimab, bimekizumab, golimumab, guselkumab, and risankizumab [32]. With emerging therapies, a decrease in morbidity due to HS may eventually be on the horizon. 

### 3.4. Emerging Findings

The treatment of HS remains a serious concern for physicians. Numerous studies are investigating effective and tolerable treatment options for HS patients to improve HS management and reduce the risk of comorbidities. These options include local excision, deroofing and curettage, laser ablation (CO_2_), laser hair removal, and even radiation. See Table 4 for a summary of seven selected articles reporting emerging findings in hidradenitis suppurativa research and practice.

Hidradenitis suppurativa assessment tools typically consider draining fistulas when assigning disease severity [41]. The fistulas are largely a debilitating aspect of HS, and patients with draining fistulas report a lower quality of life [41]. Thus, treatment of draining fistulas remains a top priority for investigators. In a recent study, the use of hypertonic saline (HTS) injection in the treatment of HS fistulas found that drainage, erythema, and swelling significantly improved from baseline [41]. HTS has also improved dermatology life quality index scores and physician-assessed overall HS improvement. Moreover, the recurrent painful nodules/abscesses that manifest with HS can be quite bothersome. Current treatment options seek ways to reduce the number of abscess nodules and improve the quality of life in HS patients [42,43,44]. One such treatment option is the use of adalimumab (ADA). In an open-label single-arm study evaluating the effect of long-term treatment with ADA on Japanese patients with moderate-to-severe HS, it was found that ADA improved patient quality of life, and most patients achieved HiSCR at week 12 [42]. Patients who received weekly ADA experienced flare reduction, which correlated with meaningful improvement in health-related quality of life. The investigators also noted sustained improvements in the total abscess nodules, skin pain due to HS, and modified Sartorius score (MSS) [42,43,44]. 

Current research seeks to improve long-term disease management of HS. Intense pulsed light (IPL) treatment is an emerging therapeutic option for mild-to-moderate HS patients [45,46]. IPL allows for the destruction of inflamed hair follicles. A within-person randomized controlled trial of Hurley stage I and II patients found that patients treated with IPL had a significant reduction in MSS, suggesting that IPL may be an effective treatment option for mild-to-moderate HS [45]. A separate study investigated the utilization of a combination of IPL and radiofrequency, termed LAight^®^ therapy, in addition to first-line therapy treatment in Hurley stage I and II HS patients [46]. In a blinded, randomized controlled trial, patients who received LAight^®^ therapy and topical clindamycin 1% solution experienced a significantly higher decrease in disease severity and improvement of quality of life in comparison to patients who received topical clindamycin 1% solution monotherapy [46]. The tolerability and efficacy of the combined treatment of LAight^®^ therapy and topical clindamycin 1% solutions as first-line therapy in Hurley stage I and II HS have promising results and can sustain or increase remission after topical antibiotic treatment for long-term disease management [46,47]. Another long-term treatment for HS patients is the use of ultrasound-guided intralesional corticosteroid injections [48]. HS patients who received ultrasound-assisted intralesional infiltration of triamcinolone acetonide experienced a greater proportion of complete response in nodules, abscesses, and draining fistulas compared to non-infiltrative lesions [48]. For abscesses and draining fistulas, the Hurley stage negatively correlated with complete response [48]. Thus, ultrasound-assisted corticosteroid infiltration can be used as a useful technique for the treatment of HS lesions [48]. 

Recently, the first case of severe HS was successfully treated with oral roflumilast, a selective phosphodiesterase inhibitor. Roflumilast showed promising results in improvement of clinical HS presentation and quality-of-life measures through alteration of the expression of inflammatory mediators, such as cytokines, involved in HS pathogenesis [49]. When medical therapy fails to treat severe cases of HS, surgical excision with split-thickness skin grafts or local flap reconstructions is often necessary. However, a newer form of reconstructive surgery, described by Gierek et al., involves using a combination of acellular dermal matrix and split-thickness skin graft, known as a co-graft, for HS treatment [50]. The authors propose this method as an innovative reconstructive technique that can enhance the healing speed, as shown by the fast closure of wounds with associated good aesthetic results in two separate cases. More research needs to be conducted to determine the effectiveness of this new reconstructive method for HS [50].

A better understanding of effective treatment modalities for HS also has implications for managing HS-associated comorbidities. As numerous anecdotal evidence suggested the coexistence of both HS and RA in individual patients, a recent large-scale population-based study sought to further investigate this association [51]. The study revealed that a positive history of RA confers 1.6 times increased risk of developing HS. In addition, HS patients older than 30 years are at an increased risk of developing RA [51]. Compared to other patients with HS without RA, HS patients with comorbid RA are significantly older, suffer greater comorbidities, and have a higher prevalence of metabolic syndrome [51]. This insight into the bidirectional association between HS and RA is an important finding for physicians caring for patients with both diseases. These physicians should be aware of symptoms suggestive of HS and RA to refer their patients to a specialist at an early stage of disease manifestation [51].

## 4. Discussion

The development of HS is multifactorial and includes an interplay of lifestyle choices, bacterial colonization, viral illnesses, and abnormal immune responses. There has been significant progress in recent research regarding the pathophysiology of HS. Such research may allow us to approach both the etiology and treatment of HS with a renewed perspective.

HS pathophysiology is thought to be initiated by blockage of hair follicles, followed by follicle rupture and a subsequent inflammatory cascade [22]. Although the primary drivers of inflammation are unknown, certain molecules are known to be overexpressed in patients with HS. Elafin and SERPINS, both inhibitors of certain protein enzymes, were reported to be overexpressed in hair follicles affected by HS [22]. Additionally, inflammatory mediators IL-1B and TNF-a were found to be inducers of elafin overexpression [51]. The upregulation of the protein inhibitors in affected hair follicles, coupled with the association between elafin and inflammatory mediators, suggests a connection between keratinocyte hyperproliferation and HS inflammation. A compromised skin barrier may also play a role in HS, as an impaired epidermis cannot function correctly. Notch signaling plays a role in epidermal immunity and inflammation. Impaired notch signaling may disturb apocrine gland homeostasis, affecting the skin barrier and potentially stimulating inflammatory mediators [52]. There are different pathways involved in the pathophysiology of HS, but evidently, they lead to the characteristic inflammatory symptom.

Treatments for mild disease typically include topical treatments, such as topical clindamycin 1%, retinoids, benzyl peroxide 5–10% wash, and resorcinol [32]. Second-line treatments involve intralesional steroid injections and oral antibiotics that may involve combination antibiotics [32]. One such combination is rifampicin and clindamycin; this combination is used when oral tetracycline fails [32]. Rifampicin is a CYP-450 3A4 inducer and may result in a significant decrease in clindamycin levels; thus, this combination increases the risk of failure due to the potential of bacterial resistance and decreased drug availability [53]. The ofloxacin and clindamycin combination treatment is an alternative as well as clindamycin monotherapy [33,54]. Biologics and immunomodulators are becoming the most effective systemic therapies employed for HS. Adalimumab and infliximab are biologics that have been shown to improve skin symptoms significantly [43]. When rifampicin, clindamycin combination therapy, and adalimumab were used to treat patients with HS, all three treatments decreased the mean modified Sartorius score [34,54]. Other treatments include local excision, deroofing and curettage, laser ablation (CO_2_), laser hair removal, and radiation. Hypertonic saline injections into fistulas and combination therapy of LAight^®^ therapy and topical clindamycin 1% have also been described [41,46,48].

## 5. Conclusions

The present review provided timely updates on the diagnosis and treatment of hidradenitis suppurativa, with an added discussion of surfacing basic science findings. This study adhered to the latest PRISMA guidelines, including developing and registering the systematic review protocol. Future research and treatment strategies will require a multidisciplinary approach considering the genetic, immunological, microbiological, and environmental factors contributing to the disease’s development and progression. Strengths of our systematic review protocol include integrating multiple study designs and involving multiple authors in the search process to reduce article selection bias. Potential limitations include the lack of bias risk assessment and external validity checks of selected articles, effectively restricting data synthesis opportunities.

## Figures and Tables

**Table 1:** Summary of articles selected to review the etiology of HS (n = 9).

Author, Year	Study Type	Key Findings
Acharya, 2020	Meta-analysis, 25 studies (101,977 patients with HS and 17,194,921 controls without HS)	– Significant association between current smoking status and HS (OR, 4.26; 95% CI [3.68, 4.94]; 20 studies). In subgroup analyses, the studies performed at the hospital/clinic (OR, 5.05; 95% CI [3.96, 6.45]) and smaller studies (OR, 5.04; 95% CI [3.72, 6.85]) reported higher ORs compared with the community-based studies (OR, 3.24; 95% CI [2.83, 3.70]) and larger studies (OR, 3.61; 95% CI [2.95, 4.41]). – Significant association between ever-smoked status and HS (OR, 4.12; 95% CI [2.67, 6.37]; *I*^2^, 94%) was found. However, ex-smoking status was not higher in patients with HS (OR, 0.65; 95% CI [0.46, 0.91]).
Choi, 2020	Systematic review, 25 studies	– HS patients are four times more likely to be obese compared to the general population (random effects pooled OR: 4.022 (95% CI [2.67, 6.07], *p* < 0.001). – Weight loss from bariatric surgery may lead to HS improvement but often results in more severe malnutrition that worsens or even leads to new onset HS post-bariatric surgery.
Molinelli, 2020	Controlled clinical trial, 92 patients affected by Hurley stage I and II HS	– Patients treated with 90 mg of zinc gluconate and 30 mg of nicotinamide once daily for 90 days reported increased disease-free survival plus reduced number and mean duration of acute flares.
Phan, 2019	Systemic review, 12 studies	– Significantly higher proportion of DM in HS cases compared to healthy controls (16.1% v. 15.7%; OR: 2.17; 95% CI [1.85, 2.55]; *p* < 0.001). – A significantly higher proportion of DM was found for HS compared with healthy controls, although the effect size was attenuated compared with unadjusted analyses (OR 1.69; 95% CI [1.50, 1.91]; *p* < 0.001).
Phan, 2019	Meta-analysis, 6 case-control studies	– Significant association between adult cases of HS and metabolic syndrome (OR 1.95, 95% CI [1.31, 2.89], *p* = 0.001).
Phan, 2020	Meta-analysis, 5 case-controls studies (36,103 HS cases and 170,517 controls)	– Significant association between HS and thyroid disease (OR 1.36, 95% CI [1.13, 1.64], *I*^2^ = 78%, *p* = 0.001).
Salvador-Rodriguez, 2020	Systematic review, N = 34 patients receiving TNF-α inhibitor treatment (adalimumab = 21, infliximab = 9, etanercept = 4)	– The median delay from exposure to TNF-α inhibitor and the development of paradoxical HS was 12 months (range 1–72). – Clinical improvement and complete remission were more frequent when the TNF-α inhibitor was stopped or switched to another biological agent with a different therapeutic target rather than maintenance or change to another TNF-α inhibitor.
Tchero, 2019	Meta-analysis, 13 randomized-controlled trials	– Adalimumab, an anti-tumor necrosis factor antibody, was superior to placebo in reducing Sartorius score (SMD = −0.32, 95% CI [−0.46, −0.18], *p* < 0.0001) and pain (RR = 1.42, 95% CI [1.07, 1.90], *p* = 0.02) when given weekly. – Recommended treatments for HS include adalimumab and laser therapy.
Tugnoli, 2020	Cross-sectional study, 38 HS subjects (22 F, 16 M), without previous major psychiatric disorders	– Evidence of a significant psychiatric comorbidity in HS patients and of a strong emotional impact of the disease: psychiatric symptoms, including depression, somatic symptoms, anxiety, and insomnia, are higher than among matched controls. – Anger, emotional fragility, and low self-esteem are important psychological correlates in these patients.

**Table 2:** Summary of articles selected to review the pathophysiology of HS (n = 13).

Author, Year	Study Design	Key Findings
Almuhanna, 2021	Meta-analysis, 7 studies (200,361 HS patients, 385,599 controls)	– HS increases the risk of developing inflammatory arthritis by 3-fold (OR 3.44; 95% CI [1.92, 6.17]), spondyloarthritis by 2-fold (OR 2.10; 95% CI [1.40, 3.15]), and rheumatoid arthritis approx. 2-fold (OR 1.89; 95% CI [1.14, 3.12]).
Choi, 2020	Systemic review, 25 studies	– HS is initiated by blockage of folliculopilosebaceous units.
Crowley, 2014	Phase 2 controlled trial, N = 154	– HS patients experience comorbidities including hypertension (39.6% of 154 patients), morbid obesity (38.3%), and depression (48.1%).
de Oliveira, 2022	Meta-analysis, 3 studies (N = 51)	– HS is associated with the plugging of apocrine gland-bearing body areas, leading to follicle rupture. – HS is associated with skin regulation pathways through the upregulation of specific genes, such as PI3, TNF-α, IL-1β, IFN-γ, KRT6, KRT16, serpin-family genes, and SPRR3.
Gau, 2022	Observational study, N = 402,021	– HS patients had a 2.63-fold prevalence of renal diseases (95% CI [2.03, 3.42]).
Gau, 2022	Meta-analysis, 10 studies (N = 560,000)	– HS and psoriasis both share TNF alpha as a risk factor.
Gau, 2023	Meta-analysis, 12 studies (N = 196,757)	– HS patients have a 1.50-fold likelihood to develop asthma (95% CI [1.24, 1.81]) and a higher risk of developing atopic dermatitis (OR 4.10; 95% CI [2.06, 8.18]).
Jiménez-Gallo, 2018	Case-control study, 19 moderate-to-severe HS patients 19 controls	– HS patients have higher IL-6, IL-8, IL-10, IL-17A, and sTNF-RII.
Liu, 2018	Meta-analysis, 4 studies (N = 634)	– HLA alleles are associated with TNF inhibitors and have a role in developing AAAs in HS patients.
Phan, 2019	Meta-analysis, 5 studies (36,103 HS patients, 170,517 controls)	– HS has a significant association with thyroid disease (OR 1.36, 95% CI [1.13, 1.64], *I*^2^ = 78%, *p* = 0.001).
Phan, 2022	Meta-analysis, 6 studies	– HS patients have a higher prevalence of strokes (OR 1.74, 95% CI [1.45, 2.09]; *p* < 0.00001).
Salvador-Rodriguez, 2020	Meta-analysis, 2 studies (N = 34)	– HS involves the dysregulation of the γ-secretase/Notch pathway, resulting in secretion of IL-23 that increases IL-17 production.
Tugnoli, 2020	Cross-sectional study, 38 HS patients, 28 controls	– HS has a significant psychiatric comorbidity in GHQ-28 (*p* = 0.001), Somatic Symptoms (p = 0.002), and Anxiety and Insomnia (*p* = 0.003).

**Table 3:** Summary of articles selected to review treatment options for HS (n = 11).

Author, Year	Study Design	Key Findings
Blok, 2016	Open-label study, N = 12	– Ustekinumab resulted in Hidradenitis Suppurativa Clinical Response 50 in 47% of patients with HS.
		– The modified Sartorius score improved in 82% of HS patients treated with ustekinumab.
Delaunay, 2017	Retrospective analysis, N = 65	– Oral ofloxacin and clindamycin resulted in improvement of HS disease activity in 58.4% of patients. – Oral ofloxacin and clindamycin resulted in complete response in 33.8% of patients with HS.
Ingram, 2016	Cochrane review, 12 studies	– The Dermatology Life Quality Index (DLQI) increased by 4.0 points with 40 mg of Adalimumab given weekly in comparison to the placebo in a single study in individuals with HS. There was a 95% confidence interval (from −6·5 to −1·5 points). – In one moderate-quality study, 5 mg kg of infliximab increased the DLQI score by 8.4 points.
Kerdel, 2019	Prospective, Open-Label, Phase 2 Study, N = 20	– 65% of patients with mild-to-moderate HS treated with Apremilast achieved Hidradenitis Suppurativa Clinical Response 30 (HiSCR30). – The most common reported adverse events to Apremilast included diarrhea seen in 20%, nausea in 15%, and depression in 10% of patients.
Kimball, 2023	Randomized, placebo-controlled, double-blind phase 3 trials N = 541	– Significantly more patients in the secukinumab every 2 weeks group (*p* = 0.015) and the secukinumab every 4 weeks group (*p* = 0.0022) had a clinical response in the SUNRISE trial. – Significantly more patients in the secukinumab every 2 weeks group had a clinical response compared with the placebo group (*p* = 0.0070) in the SUNSHINE trial.
Marasca, 2019	Single-center retrospective study, 30 patients treated with rifampicin and clindamycin; 30 patients treated with adalimumab.	– Hidradenitis Suppurativa Clinical Response was achieved by 10 patients receiving rifampicin and clindamycin. – Hidradenitis Suppurativa Clinical Response was achieved by 18 patients receiving adalimumab.
Rivitti-Machado, 2022	Systematic review, 70 studies	– Effective treatment options for HS that presents with a mild presentation includes topical medication, oral antibiotics, and various lifestyle interventions. – HS that presents with moderate to severe presentation is often refractory to the above conventional treatment options, but occasional biological agents have been shown to be effective in moderate to severe HS.
Romaní, 2019	Multicenter retrospective review, N = 14	– Ustekinumab improved Dermatology Life Quality Index (DLQI) and visual analog scale of pain in 71.42% of HS patients – There were no adverse events to ustekinumab.
Shih, 2022	Systematic review and Meta-analysis, 19 studies (N = 314)	– For HS patients treated with infliximab, the pooled response rate was 83% (95% CI [0.71–0.91]). – For HS patients treated with infliximab, 2.9% had serious adverse events.
Tchero, 2019	Systematic review and Meta-analysis, 13 studies	– The most effective therapeutic regime in reducing Sartorius score and pain in comparison to placebo was Adalimumab (SMD = −0.32, 95% CI [−0.46, −0.18], *p* < 0.0001; RR = 1.42, 95% CI [1.07, 1.90], *p* = 0.02, respectively). – HS that presents late and is intractable should be treated with surgery.
Thorlacius, 2018	Case report	– A 47-year-old man diagnosed with Hurley stage III HS had a reduced visual analogue scale score and number of lesions after 12 weeks of being treated with secukinumab.

**Table 4:** Summary of articles selected to report emerging findings in HS research (n = 9).

Author, Year	Study Design	Key Findings
Gierek, 2022	Clinical trial, N = 2	– At 2.5 months after ADM + STSG co-graft, the thickness of the epidermis was 135 μm; the epidermal density was 23.07%; and thickness of the dermis was 1162 μm. – Postsurgical health-related QoL is improved by the co-graft ADM and STSG method.
Morita, 2019	Controlled trial, N = 15	– After 12 weeks of treatment with adalimumab weekly dosing, 86.7% of patients achieved clinical response. – Adalimumab weekly dosing improved clinical response of skin pain, total abscess, and inflammatory nodule count.
Morita, 2020	Controlled trial, N = 15	– After 12 weeks of weekly dosing with adalimumab, the achievement rate in HiSCR was 86.7%. – HISCR achievement rate was sustained through week 52 at 66.7%.
Porter, 2022	Controlled trial, N = 21	– HTS sclerotherapy for HS fistula resulted in significant physician-assessed overall HS improvement after 4 weeks (*p* = 0.036). – Drainage (*p* = 0.035), erythema (*p* = −0.008), and swelling (*p* = 0.025) significantly improved from baseline to final visit. – Dermatology life quality index scores significantly improved from baseline to final visit (*p* = 0.011).
Ring, 2022	Case report	– At 3 months of monotherapy with oral roflumilast 500 μg once daily, patient reported improved QoL, less intake of tramadol, and complete psoriasis clearance.
Salvador-Rodriguez, 2020	Controlled trial, N = 193	– 81.1% of nodules, 72% of abscesses, and 53.33% of draining fistulas observed complete response after 12 wks. of ultrasound-assisted intralesional infiltration of triamcinolone acetonide 40 mg/mL. – Patients who received ultrasound-assisted corticosteroid infiltration had a Hurley stage that negatively correlated with complete response for abscesses (*p* < 0.01) and draining fistulas (*p* < 0.02).
Schultheis, 2022	Randomized controlled trial, N = 88	– After 16 weeks of treatment with LAight^®^ therapy and topical clindamycin 1% solution, the ∆IHS4 was −7.2 ± 6.7 (−60.0%) which was significantly higher in magnitude than the ∆IHS4 in the control group treated with clindamycin 1% solution alone −1.8 ± 5.6 (−17.8%, *p* < 0.001).
Schultheis, 2022	Randomized controlled trial, N = 78	– 16 weeks of additional LAight^®^ therapy allowed >90% of patients who responded to therapy in period A to maintain their IHS4-response at week 32. – 60% of non-responders during period A gained IHS4-response after 16 weeks of LAight^®^ therapy.
Van der Zee, 2020	Randomized controlled trial, N = 633	– Patients who received weekly adalimumab experienced significantly lower flares compared to placebo (*p* < 0.001). – Patients who received weekly adalimumab had longer time to first flare (*p* < 0.001) and shorter flare duration (*p* = 0.0001) compared to placebo.

## Data Availability

Not applicable.

## Reprints

michaeldiaz@ufl.edu.
